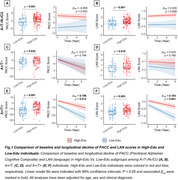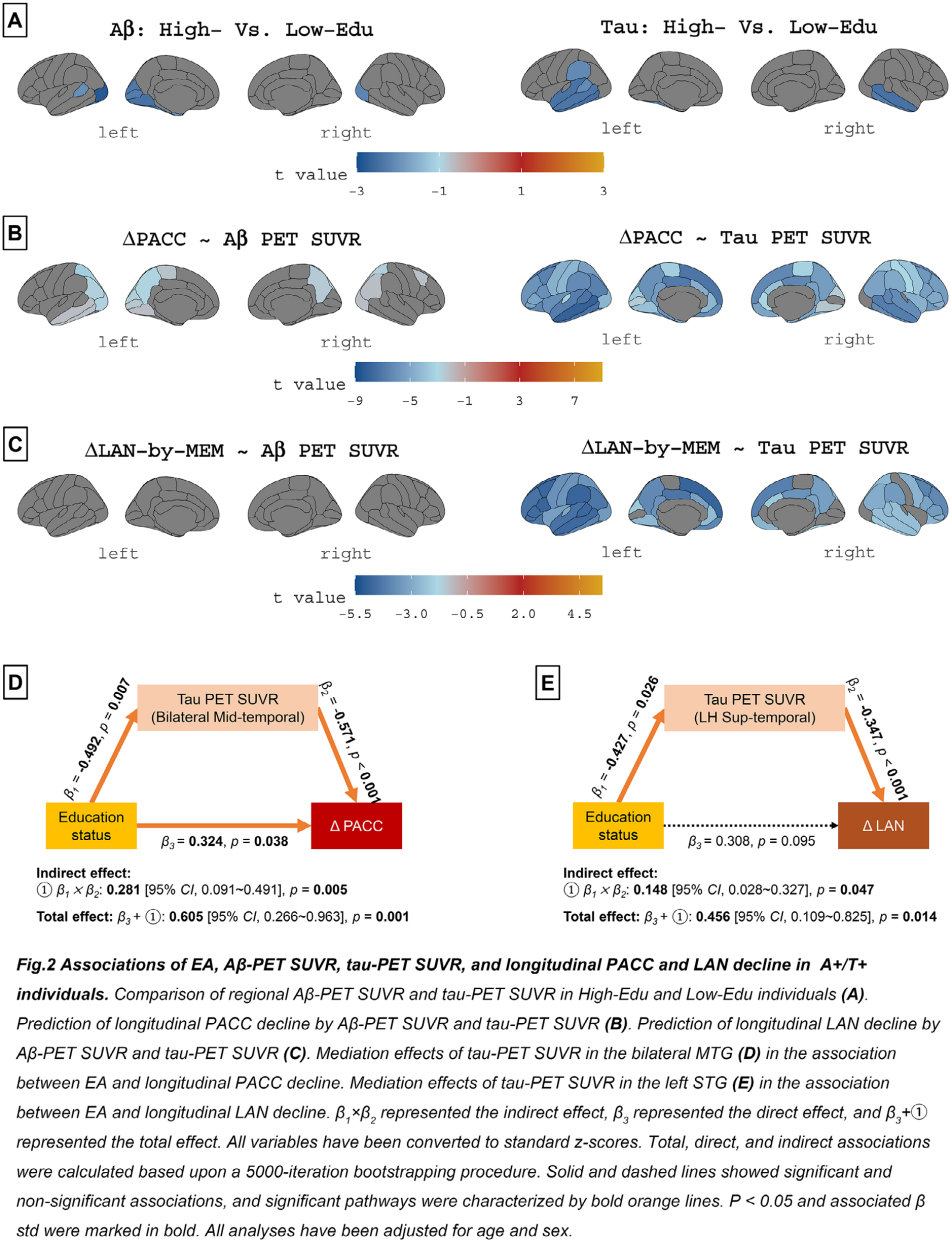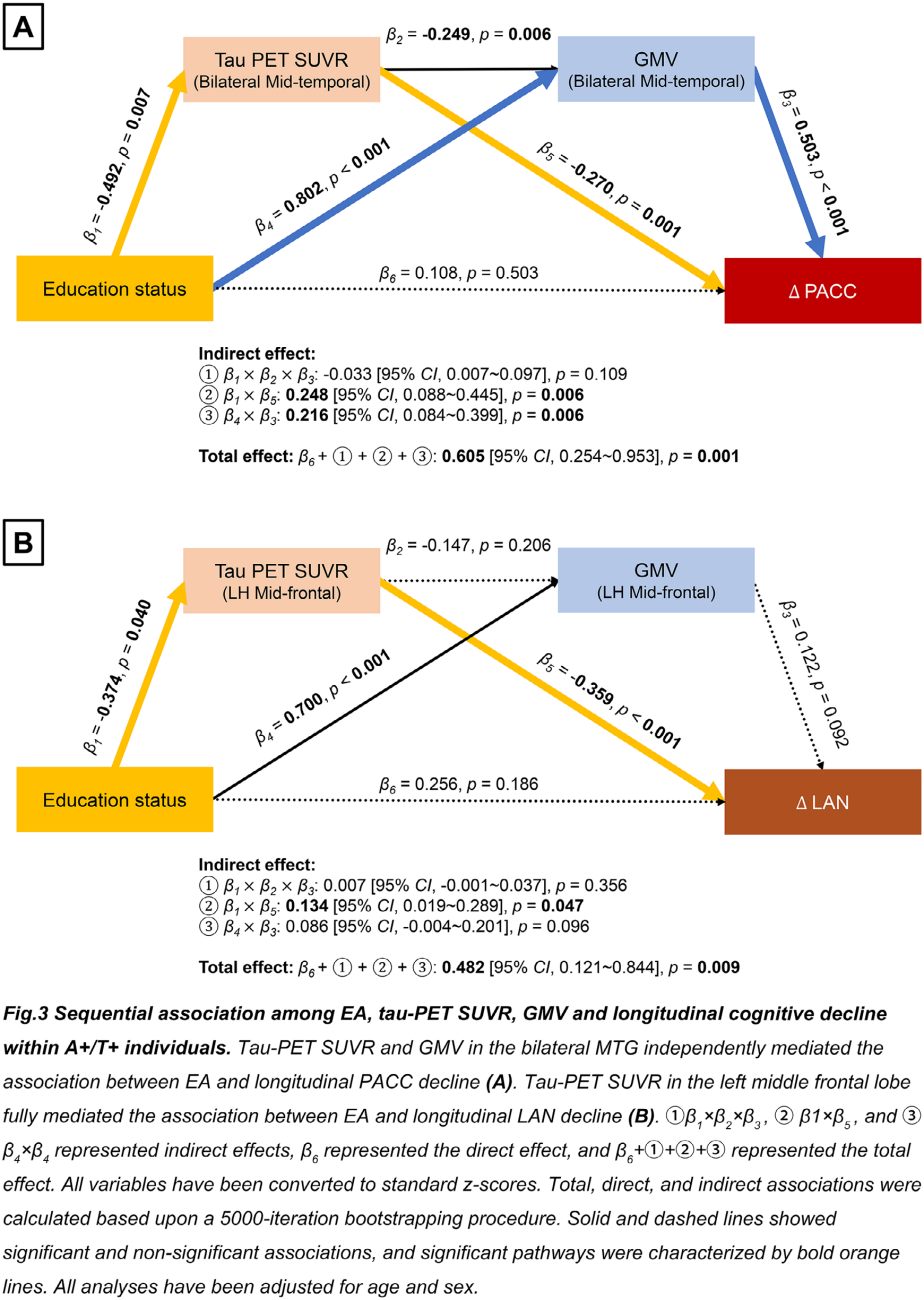# Unlocking the benefits of higher educational attainment on cognitive performance in Alzheimer’s disease

**DOI:** 10.1002/alz.091276

**Published:** 2025-01-09

**Authors:** Yue Cai, Lili Fang, Anqi Li, Xin Zhou, Jie Yang, Tengfei Guo, Alzheimer’s Disease Neuroimaging Initiative

**Affiliations:** ^1^ Institute of Biomedical Engineering, Shenzhen Bay Laboratory, Shenzhen, Guangdong China

## Abstract

**Background:**

Previous cross‐sectional studies suggest that higher educational attainment (EA) may mitigate cognitive decline in Alzheimer’s disease (AD). This study systematically investigated the association between EA and longitudinal cognitive decline across various domains and explored its connections with β‐amyloid (Aβ) plaque, tau tangle, gray matter volume (GMV) and glucose metabolism in AD.

**Method:**

We analyzed Aβ‐PET (A), tau‐PET (T), and 3D T1‐MRI images (N) from the ADNI cohort to identify 58 A+/T‐, 77 A+/T+, and 84 cognitively unimpaired (CU) participants without evidence of AD pathology and neurodegeneration (A‐/T‐/N‐/CU). All participants had longitudinal cognitive assessments covering the Preclinical Alzheimer Cognitive Composite (PACC), memory, executive function, language and visuospatial function. Additionally, a subset of 67 individuals had FDG‐PET images. Participants were divided into High‐Edu and Low‐Edu subgroups based on the median years of education of the whole cohort (16 years). Baseline and follow‐up cognitive measurements in various domains were compared between High‐Edu and Low‐Edu individuals. Furthermore, the association of EA, GMV, FDG, Aβ plaque, tau tangle, and cognitive changes were investigated.

**Result:**

Slower cognitive decline was specifically observed in PACC and language domains of the A+/T+ group among High‐Edu individuals compared to Low‐Edu individuals (Figure 1). The mediation analysis demonstrated that higher EA attenuated PACC decline through greater GMV in the middle temporal gyrus (MTG) and higher glucose metabolism in the left parahippocampal gyrus. Additionally, it mitigated language decline through greater GMV and glucose metabolism in the left inferior temporal gyrus. Furthermore, slower PACC and language decline in High‐Edu individuals was fully mediated through lower tau tangles in the MTG and left superior temporal gyrus (STG), rather than through an Aβ‐associated pathway (Figure 2). A four‐variable mediation analysis further confirmed that tau tangles and GMV in the MTG independently mediated the association between EA and PACC decline (Figure 3).

**Conclusion:**

These findings suggest that higher EA has a prominent protective effect in mitigating PACC and language decline among A+/T+ individuals. The protective effects of higher EA are mediated through the maintenance of greater GMV (or FDG) and lower tau tangles, and these pathways independently contribute to the attenuation of PACC and language decline.